# A drug-compatible and temperature-controlled microfluidic device for live-cell imaging

**DOI:** 10.1098/rsob.160156

**Published:** 2016-08-10

**Authors:** Tong Chen, Blanca Gomez-Escoda, Javier Munoz-Garcia, Julien Babic, Laurent Griscom, Pei-Yun Jenny Wu, Damien Coudreuse

**Affiliations:** 1 SyntheCell team, Institute of Genetics and Development, CNRS UMR 6290, 2 avenue du Pr. Léon Bernard, 35043 Rennes, France; 2 Genome Duplication and Maintenance team, Institute of Genetics and Development, CNRS UMR 6290, 2 avenue du Pr. Léon Bernard, 35043 Rennes, France

**Keywords:** live-cell imaging, cell biology, microfluidics, control of cellular environment, microscopy

## Abstract

Monitoring cellular responses to changes in growth conditions and perturbation of targeted pathways is integral to the investigation of biological processes. However, manipulating cells and their environment during live-cell-imaging experiments still represents a major challenge. While the coupling of microfluidics with microscopy has emerged as a powerful solution to this problem, this approach remains severely underexploited. Indeed, most microdevices rely on the polymer polydimethylsiloxane (PDMS), which strongly absorbs a variety of molecules commonly used in cell biology. This effect of the microsystems on the cellular environment hampers our capacity to accurately modulate the composition of the medium and the concentration of specific compounds within the microchips, with implications for the reliability of these experiments. To overcome this critical issue, we developed new PDMS-free microdevices dedicated to live-cell imaging that show no interference with small molecules. They also integrate a module for maintaining precise sample temperature both above and below ambient as well as for rapid temperature shifts. Importantly, changes in medium composition and temperature can be efficiently achieved within the chips while recording cell behaviour by microscopy. Compatible with different model systems, our platforms provide a versatile solution for the dynamic regulation of the cellular environment during live-cell imaging.

## 1. Background

The study of cellular behaviours and interactions has been a central focus of biologists for almost 200 years, since the initial definition of the cell as the structural and functional unit of life by Schleiden and Schwann. Recent advances in microscopy have resulted in unprecedented insights into cell physiology and have led to ever more demanding experiments for the quantitative dissection of cellular events *in vivo*. In particular, direct monitoring of cells in real time in specific and changing environments has become a valuable strategy for understanding biological processes. In this context, novel approaches using microfluidic technologies coupled with high-resolution live-cell imaging provide the possibility to study cells at both single and multicellular levels while controlling cell growth and environmental stimuli [1–5]. For instance, the combination of microfluidic systems with chemical genetics is a powerful method to selectively perturb cellular function while observing the associated physiological responses. As microchip architecture is entirely customizable, these tools can be tailored to the needs of specific cell types, co-cultures and assays [6]. This versatile technology therefore offers the potential to manipulate cells and measure their behaviour in previously unachievable ways, and represents a key turning point in our investigation of cellular mechanisms.

However, despite its vast potential in biological research, microfluidics remains underexploited in cell biological studies, due in part to the restrictions intrinsic to currently available microsystems. In the field of microfluidics for the life sciences, the design and fabrication of microchips has largely relied on the use of a transparent polymer, polydimethylsiloxane (PDMS). PDMS rapidly became the most prevalent substrate in commercially available and laboratory produced microdevices, as it possesses a number of properties that make it a material of choice: it is easy to manipulate, even outside of high-end microfabrication facilities and clean rooms, and it can be reliably bonded via plasma treatment to glass coverslips, the ideal interface for high-resolution microscopy. In addition, the possibility to deform PDMS using pressure allows the integration of valves within microsystems [7]. Nevertheless, the use of PDMS can be problematic for in-chip cell culture and manipulation [4,8–10]. Leaching of uncrosslinked oligomers, deformation of channels and cell chambers, as well as gas permeability, which can lead to evaporation and changes in medium composition and osmolarity, are some of the well-documented disadvantages of PDMS that have implications for cell physiology. Even more prohibitive is the interference of PDMS with a number of compounds: its porous and hydrophobic nature contributes to a strong absorption of small molecules and adsorption of proteins [11,12]. As previously suggested, the features that allow such molecules to cross the cell membrane—their low molecular weight and hydrophobicity—are the same as those that favour absorption by PDMS [13]. The characteristics of PDMS-based devices therefore not only preclude the accurate use of a variety of compounds in microchips, but also call into question the control of growth conditions and cellular microenvironment. Indeed, the absorption, retention and subsequent slow release of molecules make it difficult to ensure quantitative and reliable results, counterbalancing the advantages offered by microfluidic technologies. To address this often overlooked though crucial issue, a number of studies have described post-fabrication processing and treatments of PDMS devices. All of these improve the compatibility of PDMS with chemical compounds [13–16], but as we show in this study, such methods are insufficient to resolve this critical problem.

Owing to the major disadvantages associated with PDMS, the search for alternative materials for microfabrication has gained interest and urgency [8,17,18]. One group of materials with clear potential is thermoplastics, which are impermeable and show limited interaction with the chemical compounds classically used in biological studies. Thermoplastics such as polystyrene (PS) have been widely employed in conventional cell culture and are found in a range of experimental products, from tubes to Petri dishes. Importantly, they are easy to prototype with simple and affordable techniques such as hot embossing, which involves the stamping of a micropattern into a heat-softened plastic polymer [8]. In fact, a number of thermoplastics have previously been integrated in cell culture microchips, including poly(methyl methacrylate) (PMMA) [19], polycarbonate (PC) [20], cyclic olefin copolymer (COC) [21] and PS [8,22]. To date, these materials have not replaced PDMS in imaging-driven experiments, despite their purity and favourable optical properties: unlike PDMS, they cannot be easily bonded to microscopy-grade glass coverslips.

In this study, we develop a method for the simple and cost-effective fabrication of microdevices dedicated to live-cell imaging that allow for dynamic temperature control and are compatible with the use of small molecules. First, we establish a sensitive cell-based assay to demonstrate that the treatment of PDMS with previously described methods is insufficient to counteract its absorption of small hydrophobic molecules. We then identify the thermoplastic COC and paraffin wax as biocompatible materials that do not interfere with these compounds, and we construct a cell microsystem consisting of a COC chip bonded to a glass coverslip using wax as a sealant. Its fabrication does not require a complex facility, and it can be adapted to different designs of microfluidic channels, including multi-level structures. This microdevice shows no absorption of chemical compounds in our biological assays, is able to sustain cell growth and proliferation for long periods for both yeast and human cells, and is fully compatible with fluorescence microscopy. It also integrates an additional microfluidic layer that allows for dynamic control of sample temperature both above and below ambient. We further demonstrate the possibility to perform in-chip media switches as cells remain under microscopic observation. Our method therefore provides a versatile microfluidic platform for live-cell imaging and lays the foundation for new ways to investigate biological processes through modulating the cellular environment while recording real-time responses in cellular behaviour.

## 2. Material and methods

### 2.1. Fission yeast strains and methods

Standard media and methods were used [23,24]. Strains used in this study were PN1 (*972 h*^−^), DC240 (*leu1Δ:Pcdc13::cdc13-L-cdc2as::cdc13-3'UTR::ura4^+^ cdc2Δ::kanMX6 cdc13Δ::natMX6 cig1Δ::ura4^+^ cig2Δ::ura4^+^ puc1Δ::ura4^+^ ura4*−*D18 h^+^*) [25], DC450 (*leu1Δ:Pcdc13::cdc13-L-cdc2as::cdc13-3'UTR::ura4^+^ cdc2Δ:: kanMX6 cdc13Δ::natMX6 cig1Δ::hphMX6 cig2Δ::kanMX6 puc1Δ::leu2^+^ ura4*-*D18 h^+^*), MBY7519 (*Pact1::LAmGFP::leu1^+^ ade6-210 ura4*-*D18 leu1-32 h^+^*) [26], PN292 (*cdc25-22 h^+^*), PN2483 (*nda3-km311 h*^−^) and JW1001 (*ura4::eGFP::pcn1^+^ h*^−^). The *cdc2* and *cdc13* deletions as well as the Cdc13-L-Cdc2 and the Cdc13-L-Cdc2as fusion proteins were previously described [25]. Deletions of the cyclin-encoding genes *cig1*, *cig2* and *puc1* in DC450 completely remove their coding sequences. The *cdc25-22* and *nda3-km311* mutations as well as the eGFP::Pcn1/PCNA fusion were previously described [27–29]. All experiments were carried out in minimal medium plus supplements (EMM6S) at 32°C except where otherwise noted. The 3-MBPP1 and 1-NmPP1 inhibitors (A602960 and A603003, Toronto Research Chemicals Inc.) were dissolved in DMSO at stock concentrations of 10 mM and added to liquid cultures at the indicated concentrations. For cell size measurements, live cells were stained with Blankophor (MP Biochemicals) except for figure 5*a*, where DIC images were used. Cell size was determined from microscopy images using Fiji (National Institutes of Health) and the Pointpicker plug-in.

### 2.2. Mammalian cell culture

For figure 5*c*, HeLa Kyoto cell lines stably expressing an H2B::mCherry fusion protein (from plasmid 21045, Addgene) were used (Gregory Eot-Houllier 2014, unpublished data). Cells were grown in DMEM GlutaMAX (Gibco) with 10% FCS and 0.5 µg ml^−1^ puromycin. To assess the biocompatibility of the materials in our microdevices with these cells, single-channel (two ports, 300 µm wide, 40 µm high) COC/wax chips were first rinsed with growth medium, and cells were loaded at one port and allowed to fill the channels by applying a vacuum on the opposite port of the chips. The entire devices were then incubated in humid chambers at 37°C in a CO_2_ incubator, with 300 µl drops of medium deposited at each of the ports every 12 h to prevent evaporation and drying of the samples. As a control, cells were inoculated at a similar starting density in glass bottom dishes (Mat Tek Corporation) and grown at 37°C.

### 2.3. Microscopy

**Figure 1.**
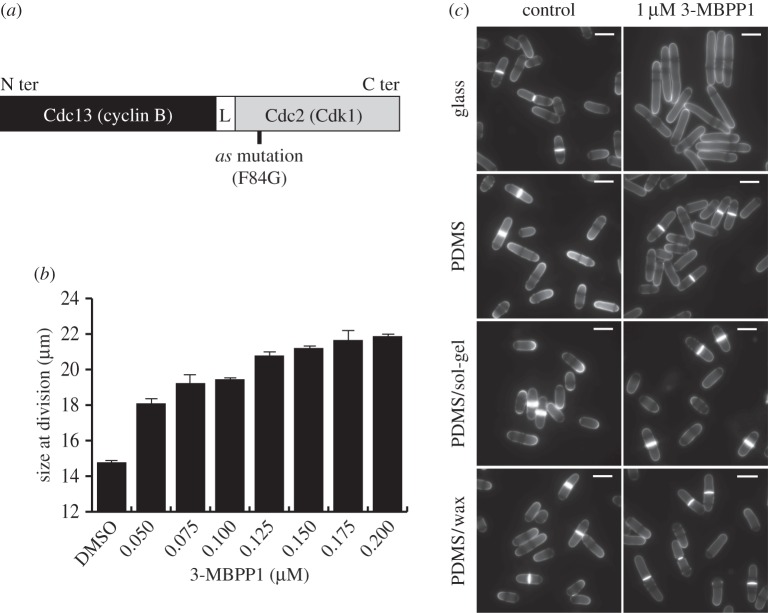
Standard treatments of PDMS are insufficient for preventing the absorption of small hydrophobic molecules. (*a*) Schematic representation of the Cdc13-L-Cdc2 fusion protein driving the fission yeast cell cycle in the strains used in this study (L = linker). The presence of the F84G mutation in the Cdc2 moiety of the protein renders the system sensitive to inhibition by characterized ATP analogues. *as*: analogue sensitive. (*b*) Cell size at division reflects the concentration of inhibitor to which cells are exposed. Batch cultures of cells operating with the inhibitor-sensitive cell cycle control module (Cdc13-L-Cdc2as fusion protein) were incubated with the indicated concentrations of 3-MBPP1 for 2 h 40 min at 32°C, and cell size at division was measured. Bars represent standard errors of three independent experiments (*n* > 50 for each independent experiment). (*c*) Drop assays were performed with inhibitor-sensitive cells on the indicated materials in the presence of 1 µM 3-MBPP1 for 2 h 40 min at 32°C. On glass, this resulted in G2 arrest with elongated, non-dividing cells. By contrast, cells grown on PDMS continued to divide, highlighting the strong absorption of such molecules by this polymer. Importantly, treatments of PDMS previously shown to limit absorption using fluorescent dye tests were revealed by this assay to be insufficient to prevent absorption, as cells kept dividing in the presence of the inhibitor. Blankophor staining. Scale bars, 10 µm.

**Figure 2.**
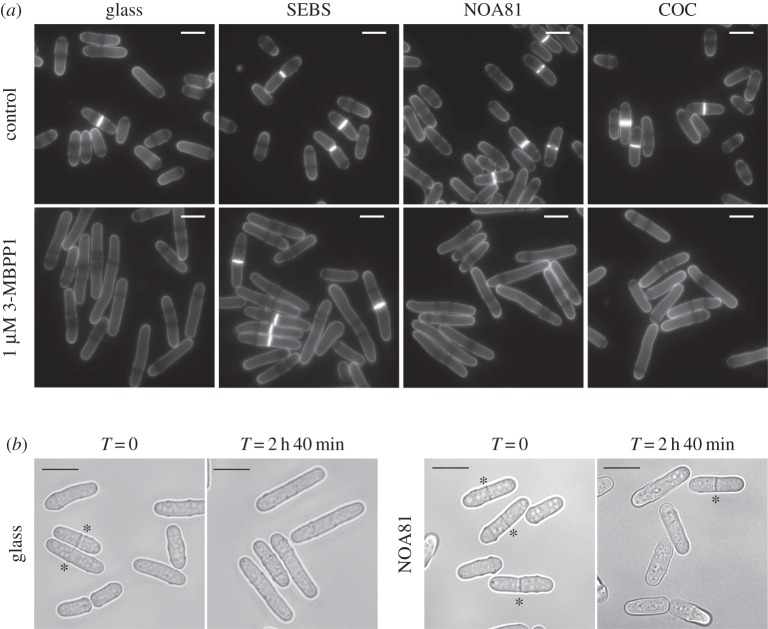
Identification of alternative materials for microfabrication. (*a*) Drop assays using inhibitor-sensitive cells on the indicated materials in the presence of 1 µM 3-MBPP1 for 2 h 40 min at 32°C. While cells on SEBS kept dividing, although at a longer size, cells on glass, NOA81 and COC showed a complete arrest of the cell cycle, suggesting no absorption of the molecule. Blankophor staining. Scale bars, 10 µm. (*b*) Inhibitor-sensitive cells were grown in microfluidic channels made of NOA81 in the presence of 1 µM 3-MBPP1 for 2 h 40 min at 32°C. In contrast with the experiment in (*a*), these cells kept dividing despite the presence of the compound, showing its significant absorption by NOA81 at a high surface area to volume ratio. Asterisks indicate septated (dividing) cells. DIC images. Scale bars, 10 µm.

**Figure 3.**
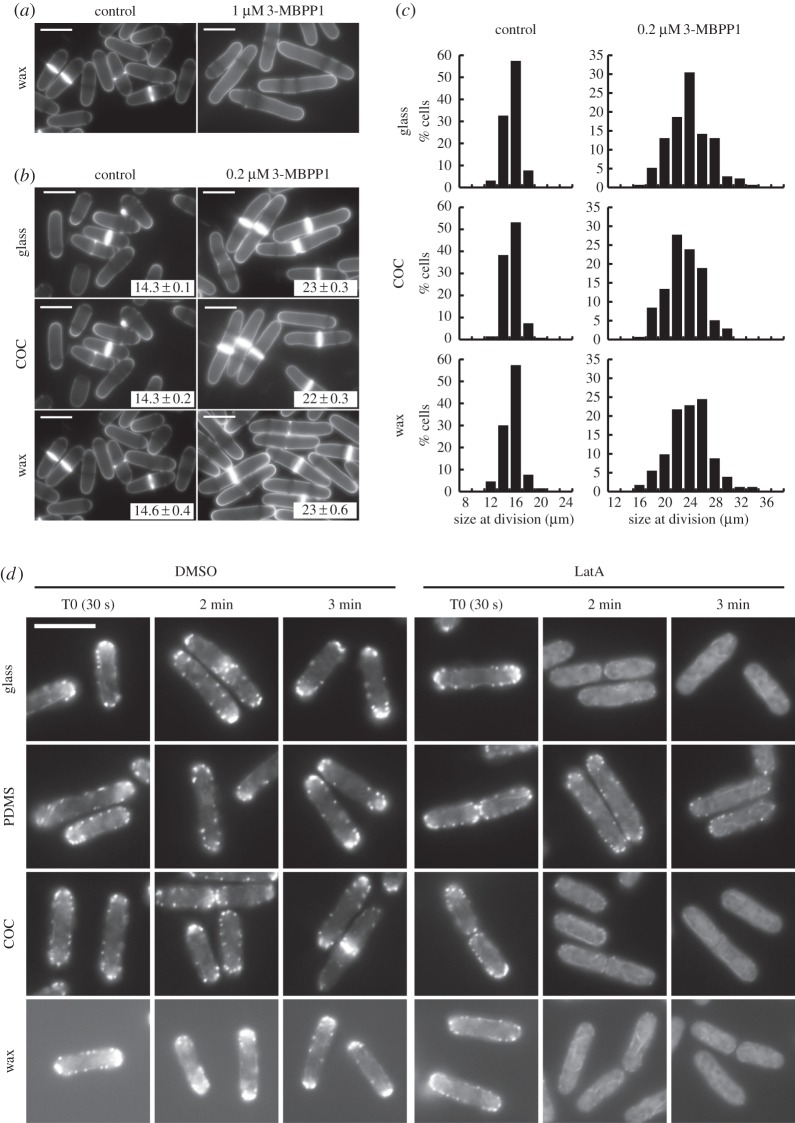
Paraffin wax as a sealant for bonding COC chips to glass coverslips. (*a*) Inhibitor-sensitive cells grown on a wax layer in the presence of 1 µM 3-MBPP1 behaved similarly to the glass control (drop assay, 2 h 40 min at 32°C, cf. figure 2*a*). Blankophor staining. Scale bars, 10 µm. (*b*) To confirm the lack of absorption, drop assays were performed on glass, COC and wax with 0.2 µM 3-MBPP1 for 2 h 40 min at 32°C, and cell size at division was determined. Images show cells stained with blankophor (scale bars, 10 µm) with the size at division in insets (average of three independent experiments; *n* > 50 for each experiment; standard errors are indicated). Drop assays were used in this study for material screening purposes. While they allow for rapid identification of promising materials, they show a higher variability than batch cultures or chip assays with regard to cell size at division. The difference observed between glass, COC and wax in this assay was not considered to be significant. This was further demonstrated by the results of figure 5*a*. (*c*) Size distribution in the populations of cells grown on glass, COC and wax in the presence of 0.2 µM 3-MBPP1 for 2 h 40 min at 32°C. Data are from the drop assays in figure 3*b* (includes all measurements from the three independent experiments in each assay). No differences were observed between the different materials. (*d*) Cells expressing the F-actin marker LAGFP [26,30] were treated with 50 µM LatA glass, PDMS, COC and wax and monitored by live-cell imaging (see Material and methods section for the specifics of the LatA assay). After 2 min, patches of F-actin were only visible in cells grown on PDMS. The stronger background fluorescence for the wax assay resulted from the poor optical properties of wax. Scale bar, 10 µm.

**Figure 4.**
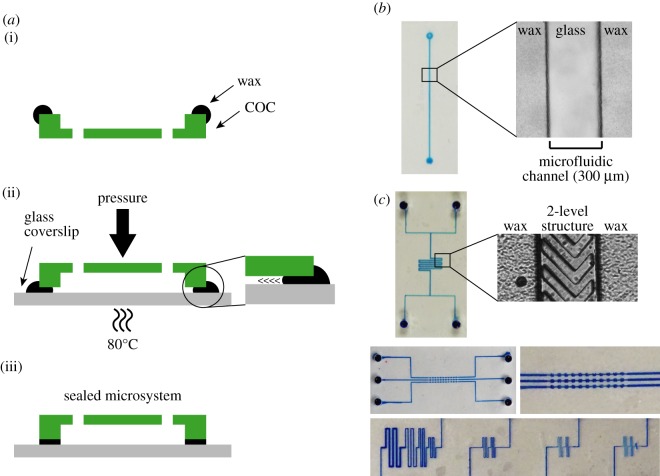
Fabrication of closed COC/wax microchips. (*a*) Schematic representation of the different steps of the wax-bonding protocol. (i) Pre-melted wax is deposited around the edge of the chip and allowed to rapidly harden at room temperature. (ii) The microsystem is then mounted on a glass coverslip and put under pressure on a hot plate at 80°C, allowing the wax to melt and spread between the COC chip and the glass coverslip. (iii) The microsystem is cooled to room temperature, generating a strongly sealed chip. (*b*) A single-channel (two ports, 300 µm wide and 40 µm high) microchip was mounted on a glass coverslip with the wax protocol. The flow of melted wax between the COC chip and the glass coverslip stopped when it encountered microchannels. Left: a bromophenol blue solution was injected for visualization. Right: microscopy image of the channel (transmitted light) showing the wax bonding. (*c*) The limits of the wax-bonding protocol were tested for channels of various sizes and structures as well as for multi-level devices (see the electronic supplementary material, figure S2, for detailed schematics of the test designs). In total, 40 µm in width and height were identified as the lower limits for the dimensions of channels required for the reliable bonding of the COC chips. Smaller structures could be achieved but with lower success rates.

**Figure 5.**
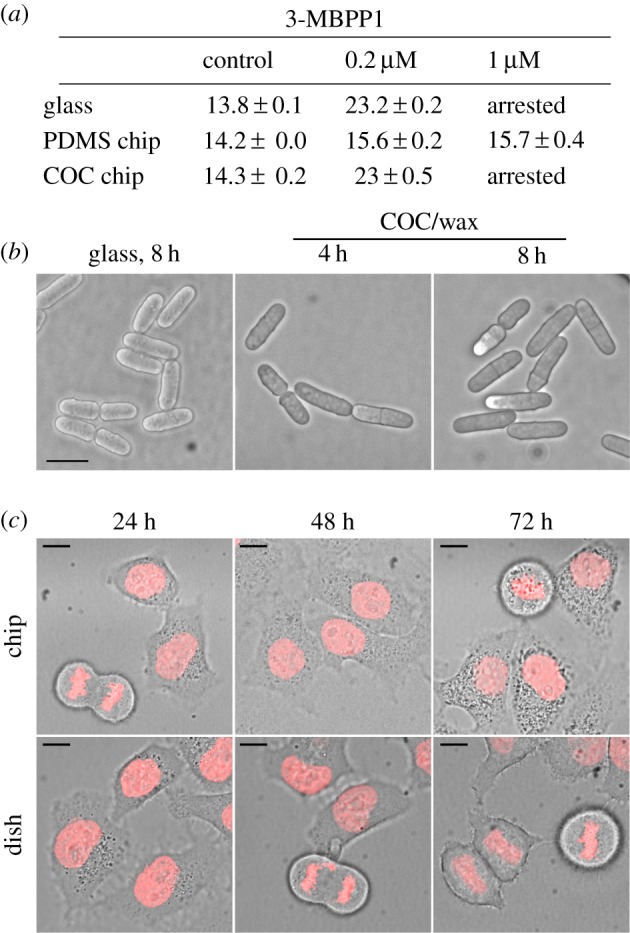
COC/wax microsystems are compatible with small molecules and can be used with different model systems. (*a*) Size at division (in micrometres) of inhibitor-sensitive cells grown at 32°C for 2 h 40 min in the presence of different concentrations of 3-MBPP1 in full PDMS or COC/wax microchips or between two glass coverslips. Average of three independent experiments with standard errors (*n* > 50 for each experiment). Identical results were obtained for glass and the COC/wax device, while PDMS showed strong absorption of the inhibitor. (*b*) Inhibitor-sensitive cells were grown in a closed COC/wax microchip at 32°C for 8 h. No defects in cell growth and morphology could be observed. DIC images. Scale bar, 10 µm. (*c*) HeLa cells expressing an H2B::mCherry fusion protein were grown in a COC/wax chip or in standard culture dishes for 72 h at 37°C. No differences between the two experimental set-ups were observed after 48 h, while a slight increase in the number of apoptotic cells was detected at 72 h in the chip, probably due to the very small volume of non-renewed growth medium in a gas-impermeable chamber. This could affect the pH, nutritional composition and/or partial pressure of oxygen and carbon dioxide in the medium. This assay demonstrated the possibility of performing live imaging of mammalian cells in our system for at least 48–72 h, and probably more if applying a constant flow of fresh medium through the chip to circumvent the problems mentioned above. Pictures are overlays of DIC images with maximum projections of Z stacks acquired to visualize H2B::mCherry. Scale bar, 10 µm.

All microscopy experiments were performed on an inverted Zeiss Axio Observer (Carl Zeiss Inc.) equipped with a Lumencor Spectra X illumination system (figures 1–5*b* and 7; electronic supplementary material, figures S1 and S3) or a laser bench (Visitron GmbH) and spinning disc confocal head (figure 5*c*; electronic supplementary material, figure S4). Images were acquired with a Hamamatsu Orca Flash 4.0V2 sCMOS camera through VisiView (Visitron GmbH) and subsequently analysed using Fiji.

### 2.4. Microfabrication materials

PDMS was prepared from the Sylgard 184 silicone elastomer kit (Dow Corning, USA). Styrene-ethylene/butylene-styrene (SEBS) blocks are a product of Kraton Polymer. NOA81 UV glue is a product of Norland Products Inc. (USA). COC pellets and sheets (Topas 5013) were purchased from Topas Advanced Polymers Inc. (USA). Paraffin wax (#411663) was purchased from Sigma-Aldrich (USA). Dymax UV glue is a product of Dymax Corp. (USA). Superglue is a cyanoacrylate-based glue from Loctite (Henkel, Germany). PR5 is a cyanoacrylate-based glue from 3M (USA). The double-sided adhesive tape used for the temperature control layer is ARcare 90445 from Adhesive Research Inc. (USA). Extruded PMMA for the fabrication of the manifold was purchased from Weber-Metaux (France).

### 2.5. Polydimethylsiloxane treatments, styrene-ethylene/butylene-styrene preparation and NOA81 chip fabrication

For sol-gel treatment [13], PDMS blocks were immersed in pure TEOS (Sigma-Aldrich) for 30 min under constant shaking. The treated blocks were then rapidly rinsed with ethanol followed by deionized water. They were subsequently immersed in a 4% (v/v) solution of methylamine (Sigma-Aldrich) for a minimum of 15 h, and then in water for 24 h to ensure biocompatibility [13]. For paraffin wax treatment, PDMS blocks were immersed for 5 min in pure paraffin wax melted in a glass container at 100°C, removed from the solution and allowed to cool down to room temperature [15]. For preparing SEBS layers, SEBS was dissolved in toluene (20–35%) and de-gassed under vacuum for 5–10 min. Dissolved SEBS was deposited on a glass slide and baked at 60°C for 5 h and then 95°C for 8 h [17]. Full NOA81 chips mounted on glass coverslips were fabricated as described [31].

### 2.6. Screening for materials compatible with small molecules

All the initial tests for small molecule absorption (figures 1, 2*a*, 3*a*–*c*; electronic supplementary material, figure S1) were performed using ‘drop assays’: exponentially growing cells were treated with the indicated concentrations of inhibitor and 10–20 µl drops of the cultures were immediately and directly deposited on the indicated materials. The set-ups were then incubated in humid chambers at the appropriate temperatures, and samples were taken for analyses of cell proliferation and size at division at the indicated times. For the LatA experiment in figure 3*d*, 1.5 µl of non-treated fission yeast cell cultures was deposited on the indicated substrates. LatA was then added to a final concentration of 50 µM (1.5 µl of 100 µM LatA) and coverslips were placed on the samples, allowing rapid live imaging of the effects of the drug.

### 2.7. Fabrication of polydimethylsiloxane moulds for cyclic olefin copolymer hot embossing and polydimethylsiloxane chips

PDMS moulds for hot embossing and PDMS chips were fabricated similarly by casting PDMS on SU-8 (2005 and 2050, MicroChem Corp., USA) master moulds as previously described [32]. Briefly, the master moulds were obtained by spin-coating thin layers of SU-8 (5–100 µm) on silicon wafers using a spincoater (Laurell Technologies, USA) according to manufacturer's instructions. Microstructures were then generated using high-resolution printed masks (JD phototools, UK) and 365 nm UV exposure (UV KUB 2, Kloe, France) followed by PGMEA (Sigma-Aldrich) development. The master moulds were then treated with trichloromethyl-silane (Acros Organics, Belgium) by vapour deposition (room temperature overnight under vacuum). PDMS moulds for hot embossing were produced by casting a 5 : 1 PDMS mixture on these SU-8 master moulds and curing at 75°C for 30 min followed by an additional baking step (200°C for 1 h). These moulds were used for direct hot embossing of COC at high temperatures (up to 200°C), accommodating pressures up to 1 tonne without significant deformation of the structures. PDMS chips were fabricated using a similar protocol, but with a 10 : 1 PDMS mixture and a single-step curing at 75°C for 1 h. For the mounting of closed PDMS devices, both chips and coverslips were plasma-treated for 1 min (Harrick Plasma), allowing direct and reliable bonding.

### 2.8. Fabrication of cyclic olefin copolymer chips

COC chips were fabricated using two different protocols. Initially, a thin sheet was generated from COC pellets (COC 5013) melted at 180°C under 16 MPa of pressure using a manual hydraulic press equipped with heated plates (Specac, UK). The obtained COC films of approximately 150–200 µm thickness were then placed on the PDMS moulds (see above) and hot-embossed at 180°C under 250 kPa of pressure. This allowed faithful reproduction of all the microstructures tested. A single PDMS mould could be used to fabricate several COC chips. To generate more reproducible systems, we then switched to pre-made COC sheets of accurate thicknesses (150 and 250 µm, Topas Advanced Polymers Inc., USA) in which we directly hot-embossed the microchannels as described above.

### 2.9. Bonding of cyclic olefin copolymer chips to microscopy-grade coverslips

Classical methods such as thermo-bonding or solvent-bonding were first tested but remained unreliable for achieving proper adhesion to glass and for maintaining the integrity of small features in the COC chips. Paraffin wax was selected as a sealing material because it is biocompatible with no absorptive properties (see the Results section). To assemble the COC device on a glass coverslip, melted wax (melting temperature: 65°C) was deposited on the edge of the chip and allowed to harden. The microsystem was subsequently placed on a 0.17 mm microscopy-grade coverslip that had been pre-cleaned with isopropanol and dried for 20 min at 70°C. The set-up was then put on a hot plate at 80°C at the indicated pressures for 1–2 min, allowing spreading of the melted wax by capillary action and bonding of the whole chip upon return to room temperature. The thickness of the wax layer was determined using a profilometer (KLA-Tencor Alpha-step IQ surface profilometer). For the tests of bonding strength, water was injected in a closed chip at various pressures using a 0–2 bar pressure controller (Elvesys, France).

### 2.10. Structures of the microfluidic networks used in this study

**Figure 6.**
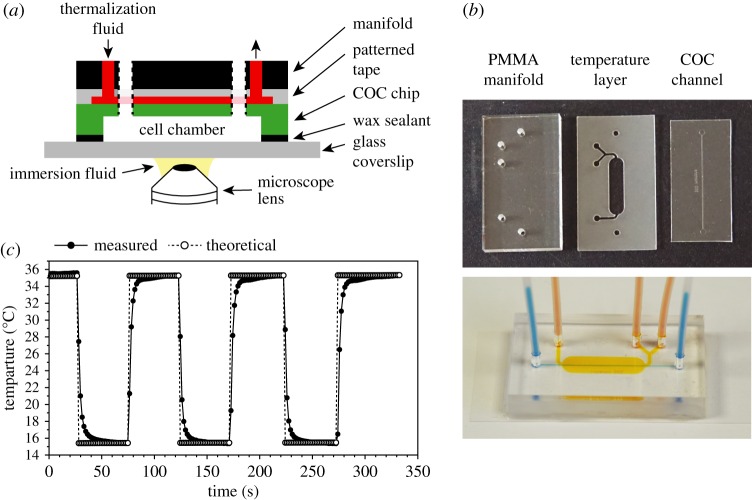
Integration of a microfluidic-based temperature control system. (*a*) Schematic representation of the full microsystem. Dashed lines represent the connections between the cell chamber and the manifold; the temperature channel is designed so that there is no fluid exchange between the cell (white) and thermalization (red) chambers. (*b*) Top panel: picture of the different elements constituting the device. Bottom panel: picture of a device mounted on a coverslip using the wax protocol. For ease of visualization, bromophenol blue and orange G dyes were injected in the cell compartment and thermalization channel, respectively. (*c*) Dynamics of temperature switches using the built-in temperature system. A COC/wax system was mounted on a glass coverslip containing temperature measurement electrodes (see Material and methods) and installed on the microscope stage, in contact with the oil of the immersion lens. The temperature within the microchannels was determined during a series of temperature shifts. The experimental data were compared with the calculated sample temperature (theoretical values) based on the measured lens and thermalization fluid temperatures at each time point and the calibration equation of the chip (similar to that provided as an example in electronic supplementary material, figure S5*a*).

**Figure 7.**
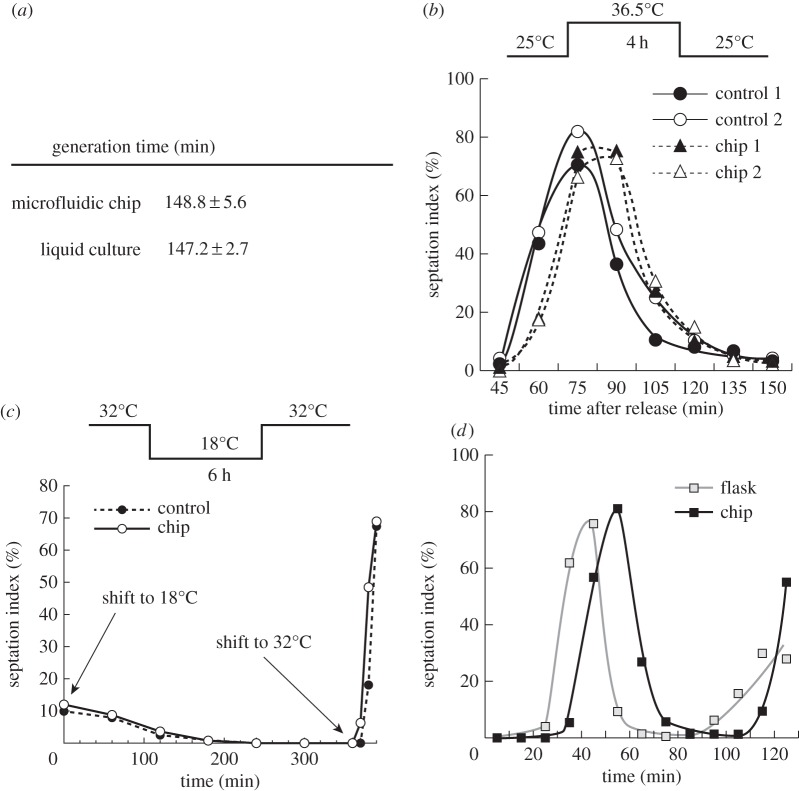
In-chip temperature and media switches. (*a*) Generation time of cells grown at 32°C in COC/wax chips with the built-in temperature control. Images were acquired every 5 min over several hours and division times were determined for 33 independent cells. Average generation time with standard deviation is shown. For the control liquid cultures, average of three experiments with standard error is shown. (*b,c*) Efficacy of the built-in thermalization system for shifting temperatures. (*b*) *cdc25-22* temperature-sensitive cells were blocked for 4 h at 36.5°C and released by shift down to 25°C using the temperature device. DIC images were acquired every 15 min, and septation index was monitored (*n* > 80 for each time point). No dividing cells were observed prior to and until 45 min after release (data not shown). (*c*) For below-ambient shifts, *nda3-km311* cold-sensitive cells were shifted from 32°C to 18°C for 6 h and released to 32°C. DIC images were acquired every hour during the 18°C block and every 10 min after release, and septation index was monitored (*n* > 100 for each time point). In (*b*) and (*c*), control cells grown in flasks were subjected to the same shifts (see Material and methods). Panel (*b*) shows two independent experiments for both chip and control. (*d*) Media switch during imaging. Analogue-sensitive cells initially grown at 32°C in batch cultures were injected in a microchip adapted to media switches (see Material and methods) and maintained at 32°C using the built-in temperature controller. Constant medium flow was applied at 40 µl min^−1^. After 1 h, medium with 1 µM 3-MBPP1 was injected for 2 h 40 min, resulting in G2 arrest. Cells were then followed for 2 h after switching back to inhibitor-free medium (*T* = 0) and septation index was determined in DIC images (*n* > 50 for each point). While cells re-entered the cell cycle with a 5–10 min delay compared with the control due to medium exchange by diffusion rather than filtration, their synchrony was similar to that in the flasks.

For figures 5*b,c* and 6*b*, and electronic supplementary material, figure S3, we used a single-channel design (two ports, 300 µm wide, 40 µm high). For figures 2*b*, 5*a*, 6*c* and 7, and electronic supplementary material, figures S4 and S5, we used a design that was compatible with the maintenance of a flow of medium through the cell compartment of the chip without flushing out non-adherent fission yeast cells. This consisted of two parallel channels (500 µm wide, 40 µm high) to accommodate the flow of medium, connected to lateral cell chambers (165 × 300 µm, 12 µm high) through small channels (15 × 100 µm, 12 µm high). A flow of medium was applied for the experiments in figure 7*a,d*.

### 2.11. Rhodamine assay

For the Rhodamine assay in the electronic supplementary material, figure S3, 50 µM Rhodamine B (Sigma-Aldrich) was injected in the microchannels and incubated for 5 min. Rhodamine B was then thoroughly washed by flowing 10 ml of water through the microsystems. Fluorescence intensity was measured by microscopy.

### 2.12. Assembly and control of the thermalization system on cyclic olefin copolymer/wax chips

The temperature control system used in our devices consists of a large chamber (7 × 16 mm) through which a flow of water pre-thermalized by Peltier elements is constantly maintained [33]; this chamber is positioned above the cell compartment (figure 6*a*). It was produced by cutting 81 µm thick double-sided tape (ARcare 90445) using a 60 W CO_2_ laser cutter (Speedy 100, Trotec, Austria). The pre-cut tape was then intercalated between the COC chip and a PMMA manifold (engineered from extruded PMMA plates of 6 mm thickness using the same laser cutter), paying particular attention to the alignment of all the ports required for the fluidic connections (figure 6*b*). The adhesion provided by the tape withstands a wide range of temperatures (10–70°C). Control of the flow rate and regulation of the temperature of the injected water was achieved using the CherryTemp system (Cherry Biotech, France). To bond the entire PMMA/temperature control/COC device to a glass coverslip, the same wax sealing protocol was used as for COC chips alone.

### 2.13. Calibration of the temperature control system

Calibration of the temperature control system required the measurement of the temperature of the sample directly within the microsystem. To this end, the complete COC microfluidic chips with the built-in temperature device were mounted on modified coverslips using the wax-bonding protocol. These consisted of standard microscopy-grade glass coverslips onto which a circuit of four electrodes linked to a resistance temperature detector (RTD) was fabricated by electrodeposition of successive metallic layers: (i) 5 nm titanium for adhesion; (ii) 70 nm platinum as a conductive element; (iii) 300 nm silicon nitride as an insulator; (iv) 2 µm Ag to reinforce the areas onto which the measurement device (model 34972A from Agilent Technologies) was connected [34].

We first calibrated the temperature detectors themselves, correlating the resistance of the RTD with its temperature, a relationship that is specific to each measurement system. To this end, the cell compartment of the chip was filled with water and a constant current was applied through two of the electrodes after precisely heating the entire microsystem to 24, 29 and 35°C (as determined by a thermistor) in incubation chambers. The voltage between the two other electrodes, which takes into account the RTD, was then measured. As the current was constant and the resistance varies linearly with the temperature, the changes in voltage directly reflected the changes in temperature.

This pre-calibrated set-up was then mounted on an inverted microscope, in contact with the oil on the immersion lens. This allowed us to determine the relationship between the temperature of the injected thermalization fluid (water, temperature measured by a PT100 sensor), the temperature of the microscope lens (measured by a second PT100 sensor) and the temperature within the microchambers (determined by the resistance of the electrodes). This was performed at 1°C intervals from 18°C to 40°C. We then established the linear equation linking the difference in temperature between the injected water and the lens with that between the sample and the lens. For a given objective temperature, this provides the exact temperature at which to set the Peltier elements in order to reach a specific target temperature in the cell compartment of any chip of the same design. Importantly, a minimum amount of oil was required to provide sufficient contact between the lens and the coverslip, and to ensure proper regulation of the temperature. Furthermore, we did not observe changes in the temperature when switching from a 63× to a 100× immersion lens (electronic supplementary material, figure S5*b*). Note that each type of thermalization fluid requires a specific calibration.

### 2.14. Temperature shift assays

For the temperature shift assays, cells were injected in the chips and the set-up was mounted on an inverted microscope. All subsequent changes in temperature (figure 7) were performed using the built-in temperature device while monitoring cell division in real time. For the control experiments with *cdc25-22* cells, batch cultures were grown at 25°C in glass flasks and shifted to 36.5°C by transfer to a pre-warmed water bath for 4 h. Release was achieved by rapidly cooling the cultures down to 25°C through shaking of the flasks in an ice/water mix prior to incubation in a 25°C water bath. For the *nda3-km311* mutant, cells grown at 32°C were shifted to the restrictive temperature of 18°C by transfer to a cooled water bath for 6 h. Release was achieved through rapid shift to a 32°C pre-warmed water bath.

## 3. Results

### 3.1. A sensitive biological readout for testing the compatibility of microfluidic systems with small molecules

A significant advantage of using microfluidics in experimental cell biology is the ability to finely and dynamically modulate the culture microenvironment, including the concentrations of specific compounds and biomolecules to which cells are exposed. In particular, the coupling of these technologies with chemical genetic strategies is poised to become a mainstream approach for the targeted regulation of cellular functions. One such method involves the specific inhibition of kinases through modifications in their catalytic pockets to accept bulky, non-reactive analogues [35–37]. These molecules bind only to the modified proteins, making possible the highly selective, dose-dependent and reversible alteration of enzyme activities. In the fission yeast *Schizosaccharomyces pombe*, we have previously engineered a system that allows for the control of cell proliferation using this technique [25]. In these cells, the Cdc2/Cdk1 protein, a conserved member of the cyclin-dependent protein kinase (CDK) family that drives both DNA replication and mitosis in fission yeast [29,38], is fused to the B-type cyclin Cdc13. This single protein module (referred to as Cdc13-L-Cdc2; L = linker) is sufficient to autonomously drive cell cycle progression. Importantly, incorporation of a mutation in the ATP binding pocket of the Cdc2 moiety of the fusion protein (Cdc2as, as: analogue sensitive) enables precise modulation of CDK activity levels through addition of non-hydrolysable ATP analogues (e.g. 1-NmPP1, 3-MBPP1) in the culture medium (figure 1*a*) [25]. Treatment with high concentrations of inhibitor results in a complete G2 block, where cells are elongated and show no nuclear or cell division [25] (also see figure 1*c*). At lower amounts, even modest alterations in inhibitor levels can be detected: small incremental increases in the applied doses of 3-MBPP1 result in cells that divide at increasingly longer sizes due to the induced mitotic delays (figure 1*b*).

In probing the possibility of using this system in the context of conventional PDMS-based chips, we evaluated the effect of the inhibitor on cells in contact with different substrates (referred to as a ‘drop assay’; see the Material and methods section). While cells treated with 1 µM 3-MBPP1 and incubated on a glass coverslip showed complete arrest in G2 phase, as expected, the same concentration of inhibitor had no impact on cells on PDMS (figure 1*c*). Absorption was also observed with 1-NmPP1 (data not shown), which we previously used to efficiently inhibit the analogue-sensitive fusion protein [25]. This demonstrates that PDMS strongly interferes with these compounds, consistent with its documented absorption of a wide variety of molecules used in biological studies [8,11,12].

As treatments of PDMS have been previously proposed to address this issue [13,15,16], we assessed their efficacy using the drop assay. We focused on the two protocols that can be performed without high-end, complex and expensive equipment, and that were validated by standard fluorescent dye-based tests for molecule absorption [13,15]. Surprisingly, we found that coating PDMS with sol-gel, which produces a glass-like surface that increases its chemical resistance [13], did not result in any detectable improvement based on our biological readout (figure 1*c*). Similarly, adding a thin layer of paraffin wax onto PDMS, which has been suggested to provide a surface that limits small molecule absorption [15], had only minor effects (figure 1*c*). We also investigated a number of undocumented alternative treatments of PDMS, from silanization of the surface to coating with UV glues, none of which led to promising results based on either biocompatibility or interference with small molecules (data not shown). Taken together, our findings highlight the sensitivity of our cell-based approach compared with conventional analyses of molecule absorption and show that well-characterized PDMS treatments fail to provide a robust solution for this problem. We therefore took advantage of our biological assay to identify alternative materials, focusing on those that may be suitable for using microfluidics in imaging-based, cell biological experiments.

### 3.2. Identification of alternative materials for microfabrication

To select new substrates for microfluidic devices compatible with live-cell microscopy, we exploited our chemical genetic system in fission yeast as a quantitative test for small molecule absorption. We chose three potential candidates based on their chemical nature, ease of manipulation, optical properties and previous reports of their integration in microdevices: (i) SEBS, a thermoplastic elastomer [17]; (ii) NOA81, a UV-glue that has been used in the microfabrication of complete NOA81/glass chips [31]; and (iii) COC, which is chemically resistant and commonly incorporated in a wide range of medical products [39]. Based on the same drop assay as above, our results demonstrated that SEBS provides a mild improvement compared with PDMS (compare figure 1*c* and figure 2*a*). By contrast, neither NOA81 nor COC exhibited small molecule interference: cells arrested in the cell cycle upon inhibitor treatment, identically to the glass control (figure 2*a*). However, while the drop assays are useful for screening purposes, they only evaluate the behaviour of cells deposited on flat substrates, at a low surface-area-to-volume ratio. This ratio is much higher in a microfluidic environment, increasing the impact of small molecule absorption by the microsystem. This prompted us to test the effect of the inhibitor within microchips in order to fully validate the selected materials. As protocols for the fabrication of complete NOA81/glass chips have previously been established [31], we assessed the absorption of 3-MBPP1 at high concentration (1 µM) in NOA81 channels. Interestingly, we found that cells continue to divide and do not arrest in the cell cycle in these conditions, thus excluding this UV-glue as a good candidate for PDMS-free microdevices (figure 2*b*). While COC remained a promising material for the fabrication of non-absorptive cell culture microsystems, similar ‘in-chip’ validation required the assembly of closed COC chips on glass. As mentioned previously, there are no methods for the reliable bonding of thermoplastic chips to glass, highlighting the need to develop a robust approach to mount such devices.

### 3.3. Construction of closed, microscopy-compatible cyclic olefin copolymer chips

Our results show that the thermoplastic COC does not interfere with small molecules in our assay, and its ability to be moulded to produce complex microfluidic networks makes it one of the best candidates for PDMS-free microchips. However, one of its limitations remains the lack of a simple method to seal it to glass coverslips, which is essential for its integration in microscopy set-ups. We therefore tested a number of glues and sealing materials, first assessing their biocompatibility using drop assays as above (electronic supplementary material, figure S1). Most of them induced cell death or changed cellular behaviour. Interestingly, paraffin wax, which has been used for the prototyping of basic microsystems [40–42], presented all the required characteristics for the fabrication of COC microdevices. First, it showed no absorption when using high concentrations of 3-MBPP1 inhibitor in drop assays (compare figure 3*a* and figure 2*a*). Moreover, at lower inhibitor concentrations, measurements of cell size at division further indicated that COC and wax are non-absorptive materials (figure 3*b,c*). We also tested the difference between glass, PDMS, COC and paraffin wax when using a fast-acting molecule with a dynamic readout that can be monitored more rapidly than that of our cell cycle assay. To this end, we treated cells with Latrunculin A (LatA), which triggers depolymerization of F actin (figure 3*d*) [26,30]. Both COC and wax behaved similarly to glass when cells were incubated with LatA. This is in contrast with cells on PDMS, where a clear delay in LatA action could be observed. Although the poor optical properties of wax render it less suitable for use as the primary material for microscopy-compatible systems, in particular in the context of multi-level devices, we hypothesized that it could serve as a sealant for mounting COC chips to glass.

To construct a closed COC microsystem on glass, we developed a method for using wax as a bonding compound. Paraffin wax melts at temperatures above 65°C, far below the transition temperature of the grade of COC (*T*_g_ = 130°C) used in our experiments. Owing to the speed with which melted wax hardens at room temperature, we found that stamping wax on the entire surface of the COC chip to seal it with a coverslip was unreliable, often leading to leaky or jammed channels. However, we reasoned that melted wax applied to the interface between the COC chip and the coverslip would spread by capillary action to bond the two surfaces. We also surmised that channels of a sufficient height and width would serve as barriers for the flow of wax, preventing it from filling the cell chambers. In our approach, small amounts of pre-melted wax were deposited on the edges of COC chips obtained by standard hot embossing (figure 4*a*; for the microfluidic networks used in this study, see Material and methods). After cooling and solidification of the wax, the systems were mounted on isopropanol-cleaned glass coverslips and placed on a hot plate at 80°C for 1–2 min under constant bonding pressure (see below). As anticipated, we observed melting and flowing of the wax between the two surfaces, with the wax front systematically stopping at the edge of the microfluidic channels (figure 4*b*). Upon return to room temperature, this adhesive layer provided strong bonding to produce a sealed microsystem (see below).

### 3.4. Physical and biological characterization of the cyclic olefin copolymer/wax chips

We next characterized the properties and potential limitations of the wax-mounted COC microsystems. First, we assessed the thickness of the intercalated wax layer as a function of the pressure applied during the assembly process (figure 4*a*). Pressures of approximately 4, 8 and 15 kPa were tested for these experiments. In all cases, the thickness of the bonding wax was in the range of a few micrometres, indicating that this technique can be applied to devices harbouring relatively fine structures (table 1). Lower bonding pressures (less than 4 kPa) increased the frequency of wax penetrating and clogging the channels, while higher bonding pressures (more than 15 kPa) led to small deformations of the COC microchannels; our results showed 4 kPa to be the most reliable. In addition, extending the time at 80°C was unnecessary and occasionally resulted in collapse of the microfluidic network. We then determined the bonding strength of the wax within the microsystems using a tensile load test. To this end, water was injected using a pressure controller in microsystems whose outputs were blocked. As a proxy for the maximum bonding strength, we evaluated the pressure required to detach the COC chip from the coverslip and generate leaks in the device (table 2). At temperatures commonly used in cell culture experiments (from 20°C to 40°C), our microchips withstood pressures up to 90 kPa, much greater than those used in standard microfluidic experiments: for instance, only 1.5 kPa was necessary to generate a high flow rate of 80 µl min^−1^ through the same test chips. Finally, we assessed the minimal size of microchannels required to delimit the spreading of melted wax during the mounting process. Using test COC chips with channels of different structures and dimensions (figure 4*c*; electronic supplementary material, figure S2), we found 40 µm to be the lower limit for both channel width and height to obtain reliable, non-obstructed microsystems. Smaller structures could also be efficiently mounted, although with a lower success rate. In addition, these assays demonstrated that our method can be used to fabricate complex microsystems, including multi-level and multi-channel chips (figure 4*c*; electronic supplementary material, figure S2). Apart from some limitations when very narrow or shallow microchannels are required, these data indicate that our platform is versatile and compatible with different applications and microfluidic circuits.

**Table 1.** Physical characterization of the COC/wax chips. The thickness of the adhesive wax layer that resulted from using various pressures for mounting the chips (figure 4*a*) was measured using a profilometer. For each applied pressure, the indicated values are means and standard deviations of independent measurements for five chips.

mounting pressure (kPa)	wax thickness (μm)
4	11 ± 1.0
8	5 ± 0.5
15	4 ± 0.5

**Table 2.** Bonding strength of the COC/wax chips. The bonding strength of the wax layer was determined by injecting medium at different pressures into microchips whose outputs were blocked. This was achieved using a high precision pressure control system (see Material and methods). ‘+’ indicates no leaks. Pressures that are incompatible with our bonding method resulted in failure of the device (‘leak’). To test the effect of temperature on the bonding strength, these experiments were performed at various temperatures using precision hot plates.

*P* (kPa)	20°C	33°C	37°C	40°C
10	+	+	+	+
15	+	+	+	+
20	+	+	+	+
25	+	+	+	+
30	+	+	+	+
35	+	+	+	+
40	+	+	+	+
45	+	+	+	+
50	+	+	+	+
55	+	+	+	+
60	+	+	+	+
65	+	+	+	+
70	+	+	+	+
75	+	+	+	+
80	+	+	+	+
85	+	+	+	+
90	+	+	+	+
95	+	+	+	leak
100	+	+	+	leak

The successful assembly of complete COC/wax devices allowed us to ascertain their absorptive properties. As mentioned above, this is particularly important because interference with any small molecule is enhanced by the high surface-area-to-volume ratio in microfluidic systems. Analogue-sensitive cells (Cdc13-L-Cdc2as) were exposed to 0.2 and 1 µM of the 3-MBPP1 within a chip for 2 h 40 min (figure 5*a*). Cells in the COC/wax chips showed the same response to the inhibitor as those grown directly on a glass coverslip. This was in contrast to the results obtained with cells in PDMS chips, which remained largely unaffected by the addition of 3-MBPP1. This difference was also confirmed using the conventional Rhodamine-based absorption assay (electronic supplementary material, figure S3). These data demonstrate that our microfluidic systems permit the use of small molecules and offer a more precise control of the environment when growing and treating cells within microdevices.

Next, we investigated the ability of our microchips to sustain long-term cell growth. To this end, we assessed the proliferation of two different model systems: fission yeast and mammalian cells. Fission yeast cells that were maintained in our microdevice at an optimal growing temperature of 32°C for 8 h (approx. three generations) showed no apparent phenotypes (figure 5*b*). Similarly, HeLa cells were observed to grow and proliferate in a wax-bonded COC chip at 37°C (figure 5*c*). Over a period of 48 h, we did not detect any differences between cells in the chip and the control culture. After 72 h, while cells in the microdevice continued to divide, there was a slight increase in the number of apoptotic cells (data not shown). While this may represent an intrinsic limitation of our device, the application of a constant flow to renew the medium in the chip may resolve this issue.

Finally, we validated our complete system for fluorescence microscopy applications. First, compared with glass, COC itself did not increase background fluorescence at the most commonly used excitation wavelengths (table 3; see glass, PDMS and COC). Second, we acquired images of cells harbouring fluorescently tagged proteins in chips and on glass coverslips. Using as a reference an eGFP-Pcn1/PCNA fusion protein, which forms discrete foci during S phase, we obtained similar results between glass, PDMS and COC/wax chips, with no detectable differences in signal to noise ratio (electronic supplementary material, figure S4).

**Table 3.** Background fluorescence in microdevices. For these measurements, culture medium was injected between two coverslips (glass), between a coverslip and a thin layer of PDMS (PDMS) or COC (COC), and within full PDMS (PDMS full) and COC/wax chips integrating the temperature control (COC full). The indicated values are means and standard deviations (more than 2000 points along a diagonal line scan). All images were acquired with a spinning disc confocal microscope (300 ms exposure at 20% laser power).

	excitation wavelengths				
	405	445	488	515	561
glass	101.1 ± 1.9	100.3 ± 1.6	102.4 ± 2.3	100.7 ± 1.8	100.6 ± 1.8
PDMS	101 ± 1.9	100.3 ± 1.7	102.3 ± 2.2	100.7 ± 1.9	100.6 ± 1.7
COC	101 ± 1.9	100.3 ± 1.8	102.2 ± 2.1	100.7 ± 1.8	100.6 ± 1.8
PDMS full	103 ± 2.5	100.4 ± 1.9	102.4 ± 2.3	100.6 ± 1.8	100.7 ± 2
COC full	103 ± 2.4	100.3 ± 1.6	102.4 ± 2.4	100.7 ± 1.8	100.6 ± 1.8

Taken together, these experiments demonstrate the advantages of our COC/wax chips, which are compatible with small compounds, provide a reliable control of the cell culture conditions and are adapted for live-cell imaging studies using different biological models.

### 3.5. Implementation of a thermalization layer

While our microsystem allows for the regulation of different aspects of the cell environment, it is also critical to control the temperature of the biological sample during microscopy experiments. Although there are a number of methods to achieve this, including incubation chambers and heated platforms, few are compatible with the rapid changes in temperature necessary for the use of temperature-sensitive mutations and with experiments that must be performed below-ambient temperature. We therefore extended our COC/wax device, adding a built-in, microfluidic-based temperature control unit. This system consists of a miniaturized thermalization chamber mounted above the cell culture channels (figure 6*a*) that accommodates a flow of water cooled or heated to a target temperature by Peltier elements [33]. This layer was produced by cutting channels in a sheet of double-sided medical adhesive (figure 6*b*; see Material and methods), and it was intercalated between the COC chip and a micromanifold made of PMMA (figure 6*a,b*). To favour heat exchange between the thermalization and sample compartments, we limited the thickness of the COC layer above the cell culture channels to 200 µm (see Material and methods). These additional elements did not increase the fluorescence background (table 3, COC full).

For accurate control of the sample temperature, it is crucial to account for the heat exchanges that occur between the device and its surroundings, in particular through contact between the coverslip and the microscope objective when using an immersion lens. We therefore calibrated our system by establishing the relationships between (i) the temperature of the water set by the Peltier elements, (ii) the temperature of the microscope lens in contact with the device through the immersion fluid and (iii) the temperature of the sample [33] (electronic supplementary material, figure S5*a*). A detailed description of the calibration protocol is presented in the Material and methods section. It integrates metal electrodes that are deposited on the microsystem's coverslip, allowing for direct measurements of the temperature in the cell chambers. For a given lens temperature, this calibration establishes the temperature that must be imposed by the Peltier elements in order to achieve the proper target temperature in the cell channels. We noted that switching the lens had no effect on the sample temperature (electronic supplementary material, figure S5*b*), suggesting that the use of different objectives does not require specific calibrations. Importantly, once this procedure has been performed for a microsystem, it remains valid for any chip of the same design. Next, we assessed the capacity of our microdevice to produce rapid and accurate temperature switches. Taking advantage of the same electrodes as those used for the calibration procedure, we measured the kinetics and reproducibility of the shifts. Sample temperature was within 0.5°C of the target temperature in 10–15 s when shifting down and in 15–25 s when shifting up (figure 6*c*). These results showed that our microchip allows for tight and dynamic control of the temperature of the sample at the microscope.

### 3.6. Temperature and medium control in a biological context

The data presented so far suggest that our microdevice could be a powerful platform for controlling the cellular environment during live-cell imaging experiments, and we validated its use in a biological context. First, we tested the ability of the system to maintain a constant temperature, taking advantage of the well-characterized generation time of fission yeast cells in minimal medium at 32°C. The division time of cells grown in the chips was determined by live-cell imaging and compared with standard batch cultures. To prevent any potential nutritional bias, a constant flow of fresh medium was applied throughout the experiment (10 µl min^−1^; at this flow rate, we observed no influence of the injection of medium on the temperature of the sample; see electronic supplementary material, figure S5*c*). This required the use of a specific chamber design in which non-adherent cells are maintained outside of the direct path of the flow of medium (see Material and methods). Our results showed identical cell cycle times between the two approaches, validating the stability of our system for sustaining a given temperature (figure 7*a*).

To evaluate the performance of the temperature shift, we then conducted experiments using temperature-sensitive fission yeast mutants (figure 7*b,c*). The *cdc25-22* mutation [29] inactivates the Cdc25 phosphatase, required for entry into mitosis [43], at a restrictive temperature of 36.5°C. *cdc25-22* cells proliferate at the permissive temperature of 25°C, and shifting to 36.5°C results in G2 arrest and cell elongation. After 4 h at restrictive temperature, a shift back to the permissive temperature allows for the activation of the mitotic machinery and synchronous cell cycle re-entry [44]. In addition, to test the use of the system for temperatures below ambient, we assayed a cold-sensitive mutation of *nda3*, which encodes for beta tubulin. The *nda3-km311* mutant proliferates normally at 32°C, while growth at 18°C results in a mitotic block [27]. After 6 h at the restrictive temperature, a shift back to 32°C results in synchronous progression through mitosis and the subsequent cell cycle phases. When these strains were subjected to the appropriate temperature shifts in the microfluidic chips using the integrated temperature control system, we found that the timing and synchrony of cell cycle progression were similar to standard experiments performed with batch cultures grown in glass flasks (figure 7*b,c*). These analyses demonstrate the ability of our device to generate rapid and accurate changes in temperature both above and below ambient. This not only enables the use of heat- and cold-sensitive mutations during live-cell imaging but also makes possible the assessment of temperature-sensitive processes in real time.

Finally, we demonstrated that our system allows for in-chip media switches, using treatment with 3-MBPP1 as an assay. Inhibitor block followed by release into normal medium in batch cultures is a well-characterized method for obtaining synchronous populations of cells [25]. Cells initially maintained at 32°C with a constant flow rate of fresh medium within the chip were arrested for 2 h 40 min by switching to medium containing 1 µM inhibitor. As expected, this resulted in complete cell cycle arrest, with cells elongating without undergoing mitosis. Switching back to inhibitor-free medium resulted in synchronous entry into the cell cycle (figure 7*d*). A small delay in this process was observed when using the chip, resulting from the time required for medium exchange by diffusion compared with filtration in the control cultures. This is inherent to microfluidic devices designed to avoid mechanical and shear stresses that can be induced if cells are maintained directly in the medium flow. However, the degree of synchrony was identical to that obtained when filtering batch cultures. Importantly, all the different shifts were performed while cells were under microscopic observation. Taken together, these results demonstrate that our microsystem allows for the dynamic control of both the temperature and medium composition during live-cell-imaging experiments. Coupling various media and temperature changes represents a powerful approach to study complex processes at the cell biological level.

## 4. Discussion

The introduction of microfluidic approaches in the life sciences has resulted in exciting possibilities for the investigation of cellular behaviour. By allowing for the control of cell growth and external stimuli at high temporal and spatial resolution, these methodologies pave the way for a new quantitative dimension in cell biology. One essential aspect of these technologies is the ability to reproducibly manipulate the cellular environment in customized microsystems. Paradoxically, the most widely used material for microfluidics, PDMS, has been shown to interfere with a variety of compounds. This is particularly true for small hydrophobic molecules, a class that includes a large number of cell biological reagents. For microscopy-based experiments, no effective solutions were available. In this context, finding alternatives to PDMS-based microsystems has become essential for exploiting the full potential of microfluidic devices. In this study, we have used one such small hydrophobic molecule, the ATP analogue 3-MBPP1, as a model compound to establish a sensitive and quantitative cell-based assay for absorption. This enabled us to assess the properties of potential microfabrication materials and identify appropriate substrates for PDMS-free microsystems compatible with high-resolution microscopy. We developed a new approach to produce microfluidic devices for live-cell imaging in which temperature and medium composition can be dynamically modulated. Our method is relatively cost-effective, and does not require high-end equipment and facilities. Importantly, it accommodates the design of custom networks of channels and can potentially be applied to bond any rigid microdevice to glass. This versatile tool therefore makes it possible to perform reproducible, highly controlled quantitative experiments in a broad range of contexts where cellular behaviour and responses to external stimuli must be monitored in real time.

## Supplementary Material

Supplementary Material

## Data accessibility

The datasets supporting this article have been uploaded as part of the electronic supplementary material.
