# Supplementary material for: A drug-compatible and temperature-controlled microfluidic device for live-cell imaging
Source: Open Biol. 2016 Aug 10;6(8):160156. doi: 10.1098/rsob.160156 (PMC5008015; doi:10.1098/rsob.160156)

SUPPLEMENTARY MATERIAL

Supplementary Figure 1. Identification of a sealant for COC chips.

The biocompatibility of several glues and sealing components were tested for the fabrication of closed COC chips on glass coverslips. Most of them induced strong lethality of fission yeast cells (data not shown). Drop assays (wild-type cells, 6 h at 32 °C) on representative compounds are shown here. Dymax and SuperGlue were not biocompatible. While the PR5 compound had no negative impact on cell proliferation, cells appeared to adhere to the glue. Paraffin wax, however, did not have detectable side effects (compare to Glass in Figure 2a). Blankophor staining. Scale bars = 10 µm. (PDF).

Supplementary Figure 2. Limits of the wax bonding protocol.

A-C. Schematics of the designs used in Figure 4c, with all the sizes, dimensions and heights of the channels indicated. In A, the middle and right panels detail the multi-level structures of the design shown in the left panel. (PDF).

Supplementary Figure 3. Rhodamine test for small molecule absorption.

50 µM Rhodamine B was injected in PDMS and COC/Wax microchips, incubated for 5 min and washed with 10 ml water. Images were acquired before and after washing the channels (left panel). Identical microscope acquisition settings were used for each set of images for comparison between PDMS and COC. Right panel: line scans of the intensity measured through the channels (vertical scans from images in the left panel). After washing, the COC/Wax chip exposed to Rhodamine showed similar signals that filled with water. In contrast, a significantly stronger signal remained in the PDMS chip. (PDF).

Supplementary Figure 4. Compatibility of the complete device with fluorescence microscopy.

A. Fission yeast cells expressing an eGFP-Pcn1/PCNA fusion protein were imaged between standard glass slides and coverslips, in PDMS chips or in complete COC/Wax devices integrating the temperature control system. No differences were observed between the different conditions. Maximum projections of z-stacks (0.2 µm steps). Scale bar = 10 µm. **B.** Quantification of the signal and background fluorescence from the images in A. No differences in signal to noise ratio were observed between the conditions. Average nuclear fluorescence intensity was measured using maximum projections of the acquired z-stacks. n=100 for each measurement. Cells with strong foci where excluded to avoid bias in the measurements. For the background values (Bkg), quantifications were performed using similar surface areas in different regions of the images that did not contain any cells. Box-and-whiskers plot using interquartile ranges; dots represent outliers. (PDF).

Supplementary Figure 5. Characterization of the temperature control system.

A. Example of a chip calibration. Graph showing the linear relationship linking the differences in temperature 1) between the thermalization fluid (water) injected in the system (T_i) and the microscope lens (T_{lens}) and 2) between the sample (T_{sample}) and the microscope lens (T_{lens}), as determined by our calibration protocol (see Materials and Methods). The associated equation is shown, providing the temperature at which to set the Peltier elements in order to reach a specific target in the cell chamber, after measuring the temperature of the microscope lens. **B.** Impact of changing microscope lens on the temperature control system. Complete COC/Wax chips were mounted on glass coverslips with integrated temperature measurement electrodes. Fixed temperatures were applied for the thermalization fluid and the temperatures within the microdevice were monitored using

the electrodes. No differences in temperature could be detected between the 63X and 100X oil immersion objectives. **C.** Impact of flowing fresh medium at room temperature through the cell compartment of the chip on the temperature of the samples. A COC/Wax chip integrating the temperature control device was mounted on a glass coverslip integrating temperature measurement electrodes and set up on a microscope stage in contact with the objective through immersion oil. A constant temperature of 45 °C was set at the Peltier elements for the thermalization fluid. The temperature in the cell chamber was then monitored while applying different flow rates of medium. Although the highest flow rate of 60 µl/min altered the temperature of the sample, lower rates (15 and 30 µl/min) had no impact. For comparison, the total volume of a chamber of 1 cm diameter and 100 µm high is about 8 µl, which indicates that the rates allowed by the temperature system are compatible with rapid exchange of medium in the chips. (PDF).

Supplementary Figure 1

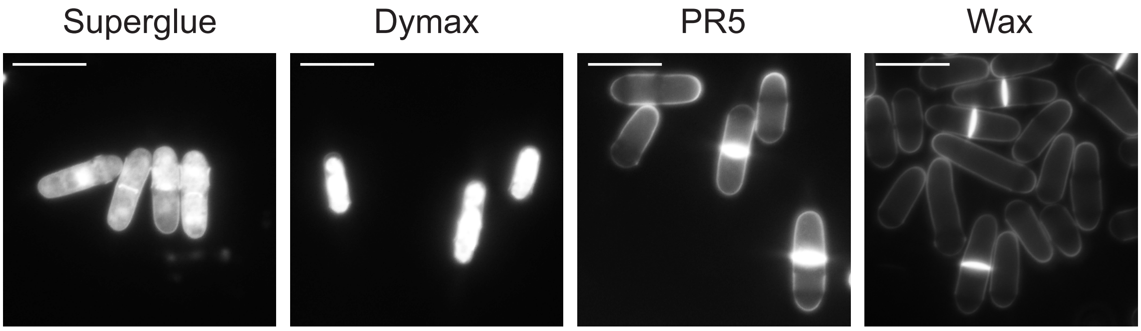

Supplementary Figure 2

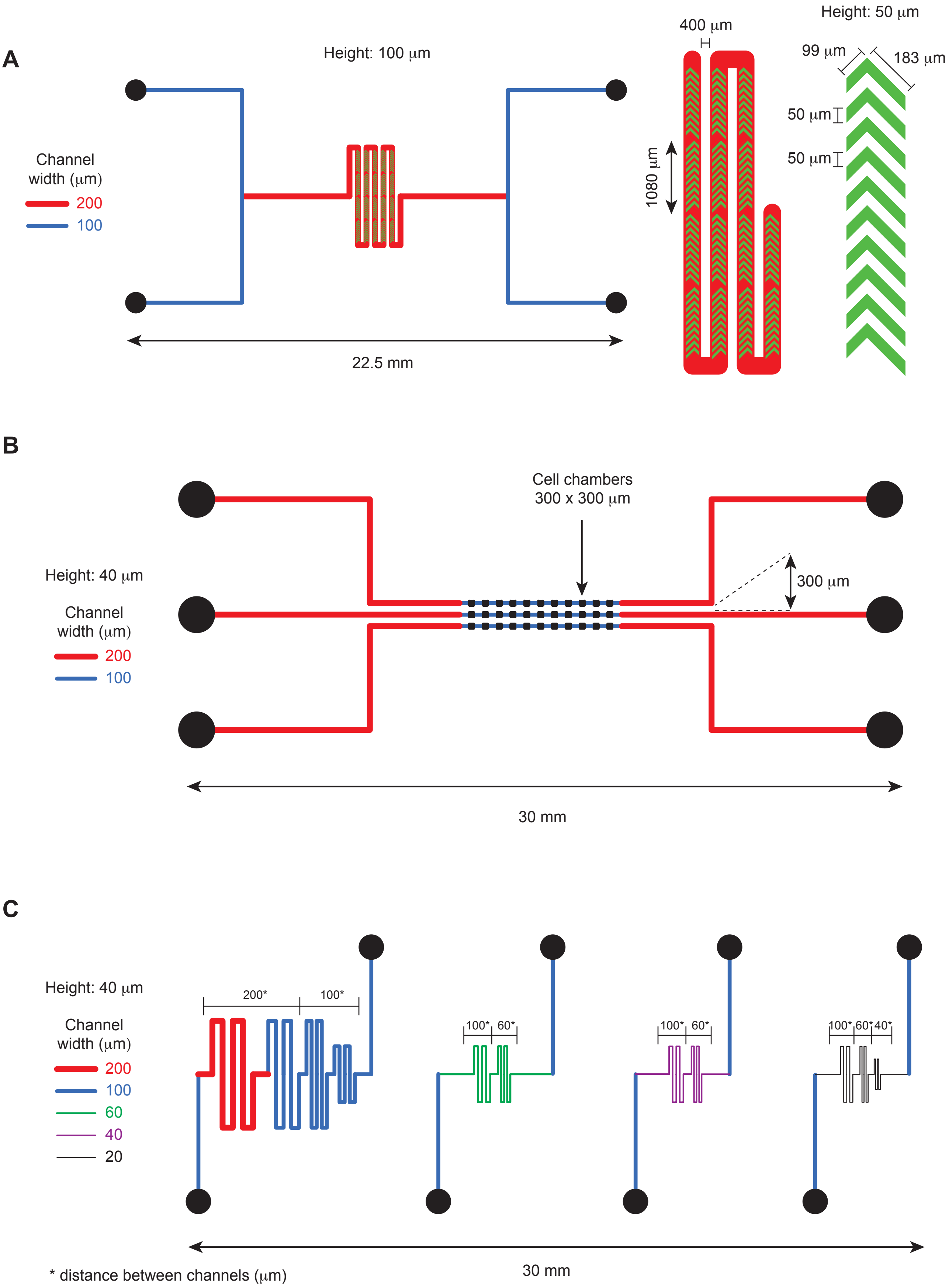

Supplementary Figure 3

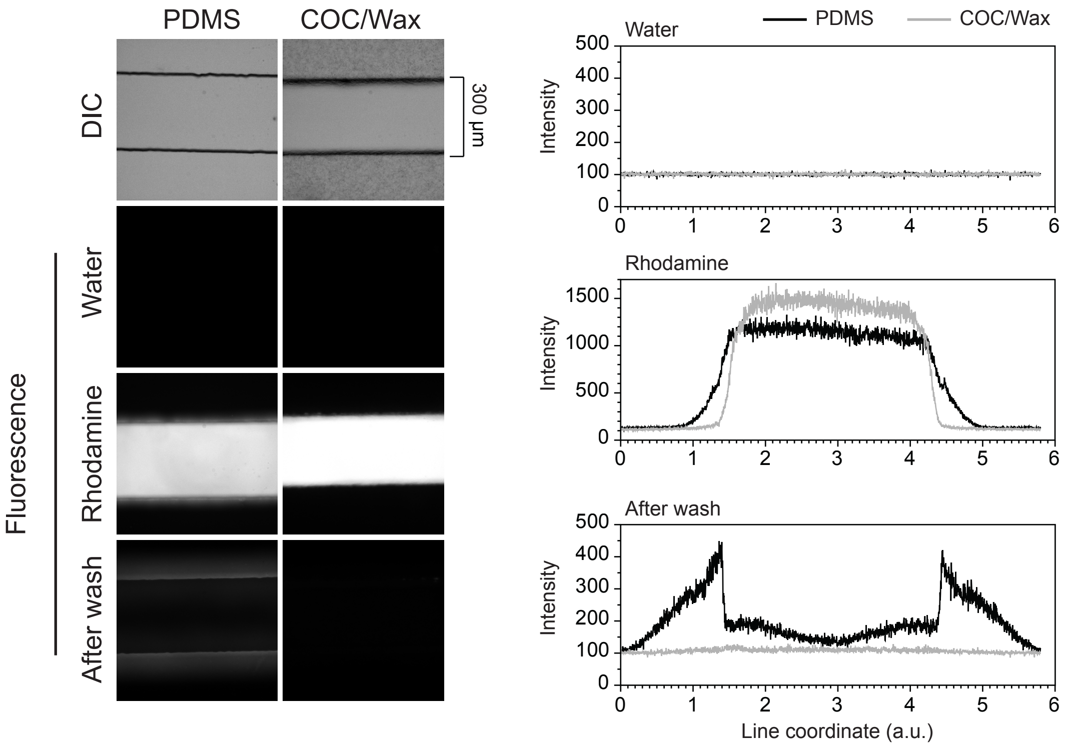

Supplementary Figure 4

A

B

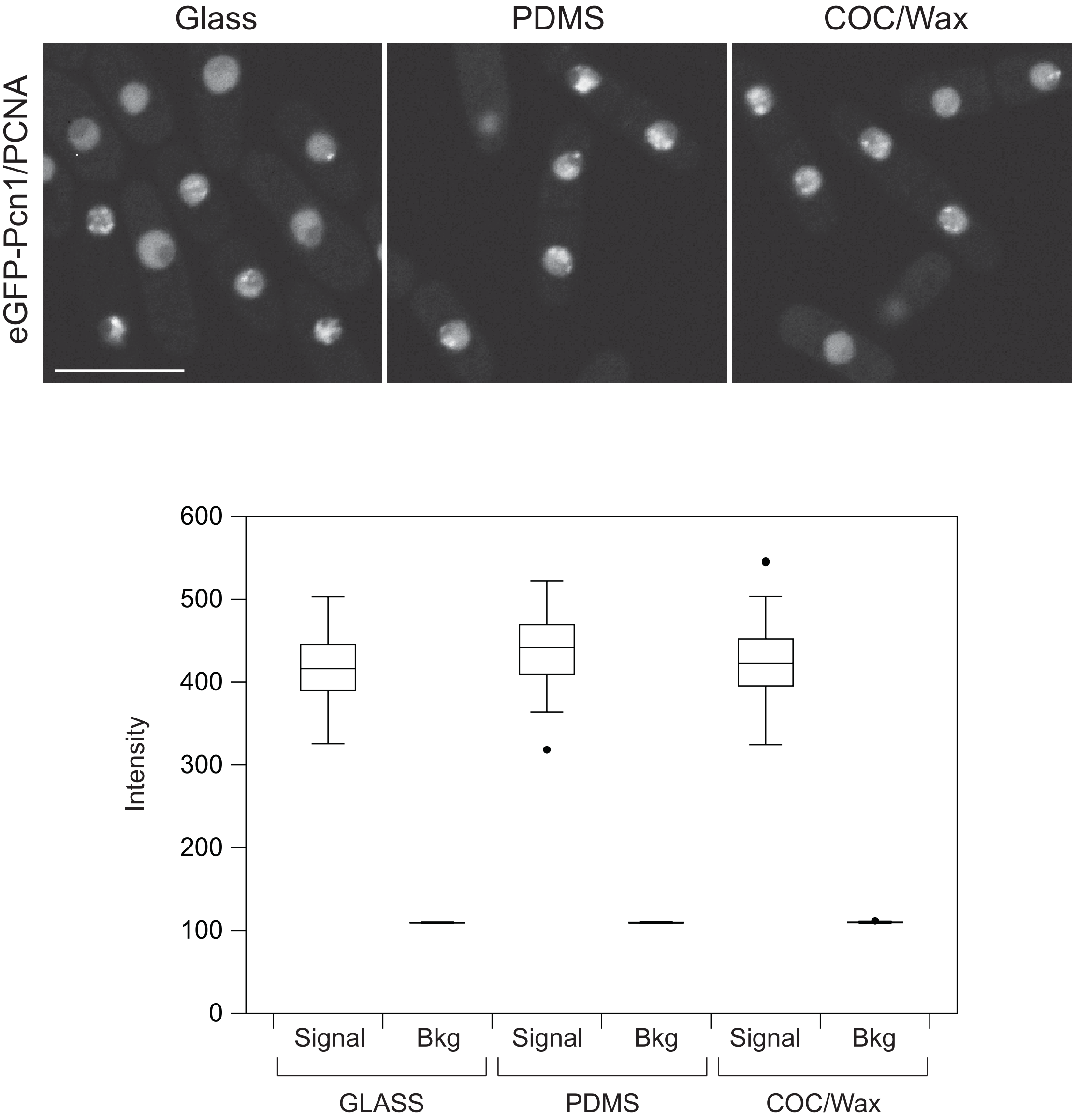

Supplementary Figure 5

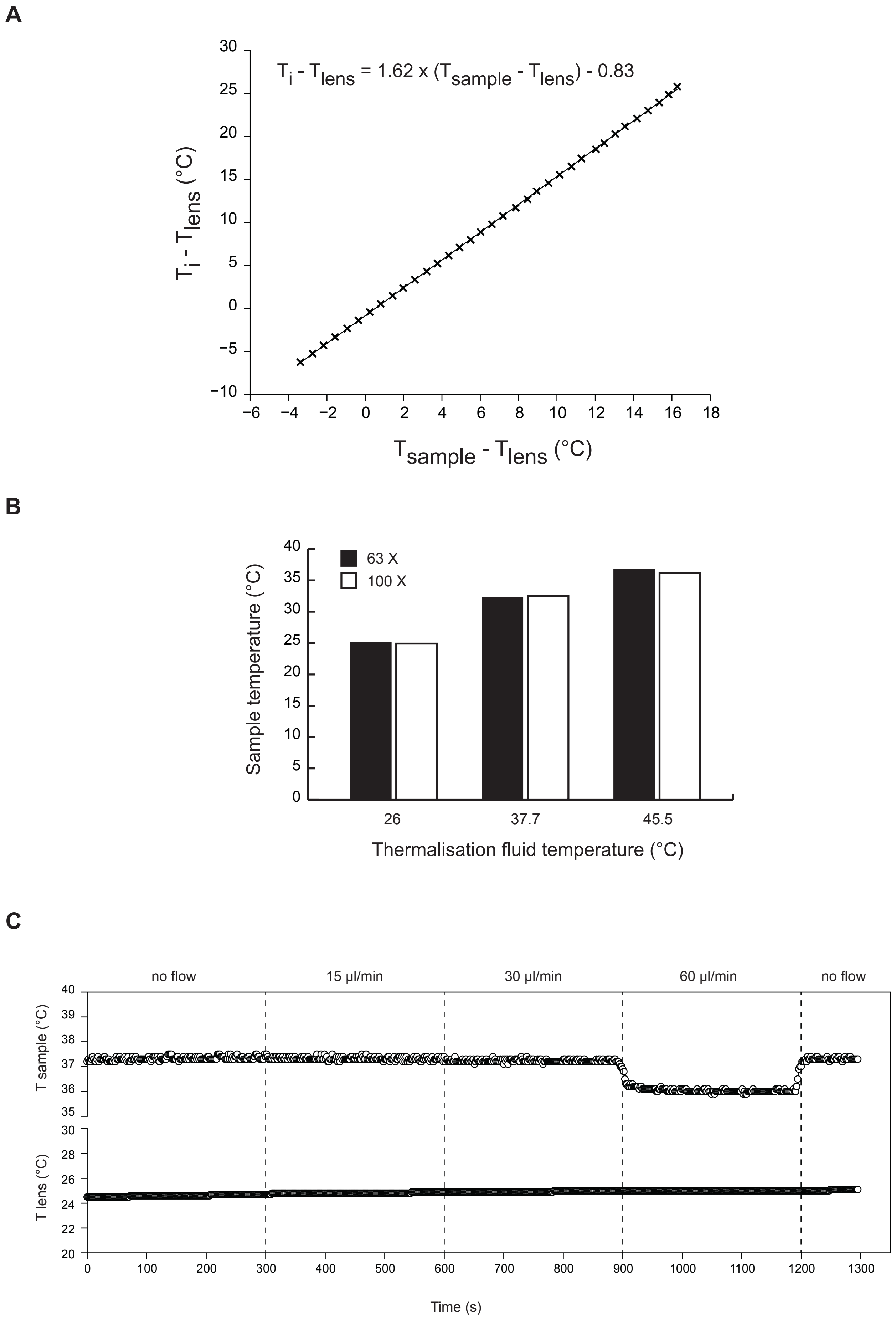

Supplement: Supplementary Material [file rsob160156supp1.pdf]
